# Mitochondrial Dysfunction in the Development and Progression of Cardiometabolic Diseases: A Narrative Review

**DOI:** 10.3390/jcm14113706

**Published:** 2025-05-25

**Authors:** Loukia Pliouta, Stamatios Lampsas, Aikaterini Kountouri, Emmanouil Korakas, John Thymis, Eva Kassi, Evangelos Oikonomou, Ignatios Ikonomidis, Vaia Lambadiari

**Affiliations:** 1 Diabetes Center, 2nd Department of Internal Medicine, Attikon University Hospital, Medical School, National and Kapodistrian University of Athens, 12462 Athens, Greece; plioutaloukia@gmail.com (L.P.); lampsas.stam@gmail.com (S.L.); katerinak90@hotmail.com (A.K.); mankor-th@hotmail.com (E.K.); vlambadiari@gmail.com (V.L.); 2 2nd Cardiology Department, Attikon University Hospital, Medical School, National and Kapodistrian University of Athens, 15772 Athens, Greece; johnythg@gmail.com; 3 Endocrine Unit, 1st Department of Propaedeutic and Internal Medicine, Laiko Hospital, National and Kapodistrian University of Athens, 11527 Athens, Greece; ekassi@med.uoa.gr; 4 3rd Department of Cardiology, “Sotiria” Thoracic Diseases Hospital of Athens, Medical School, National and Kapodistrian University of Athens, 11527 Athens, Greece; boikono@gmail.com

**Keywords:** mitochondrial dysfunction, cardiovascular disease, metabolic syndrome, mitochondrial dynamics

## Abstract

Mitochondria play a central role in energy metabolism and continuously adapt through dynamic processes such as fusion and fission. When the balance between these processes is disrupted, it can lead to mitochondrial dysfunction and increased oxidative stress, contributing to the development and progression of various cardiometabolic diseases (CMDs). Their role is crucial in diabetes mellitus (DM), since their dysfunction drives β-cell apoptosis, immune activation, and chronic inflammation through excessive ROS production, worsening endogenous insulin secretion. Moreover, sympathetic nervous system activation and altered dynamics, contribute to hypertension through oxidative stress, impaired mitophagy, endothelial dysfunction, and cardiomyocyte hypertrophy. Furthermore, the role of mitochondria is catalytic in endothelial dysfunction through excessive reactive oxygen species (ROS) production, disrupting the vascular tone, permeability, and apoptosis, while impairing antioxidant defense and promoting inflammatory processes. Mitochondrial oxidative stress, resulting from an imbalance between ROS/Reactive nitrogen species (RNS) imbalance, promotes atherosclerotic alterations and oxidative modification of oxidizing low-density lipoprotein (LDL). Mitochondrial DNA (mtDNA), situated in close proximity to the inner mitochondrial membrane where ROS are generated, is particularly susceptible to oxidative damage. ROS activate redox-sensitive inflammatory signaling pathways, notably the nuclear factor kappa B (NF-κB) pathway, leading to the transcriptional upregulation of proinflammatory cytokines, chemokines, and adhesion molecules. This proinflammatory milieu promotes endothelial activation and monocyte recruitment, thereby perpetuating local inflammation and enhancing atherogenesis. Additionally, mitochondrial disruptions in heart failure promote further ischemic injury and excessive oxidative stress release and impair ATP production and Ca^2^⁺ dysregulation, contributing to cell death, fibrosis, and decreased cardiac performance. This narrative review aims to investigate the intricate relationship between mitochondrial dysfunction and CMDs.

## 1. Introduction

Mitochondria, commonly known as “the powerhouse” of the cell, are key organelles in eukaryotic cells, vitally responsible for energy transformation, generating large amounts of ATP for cellular metabolic processes, such as the tricarboxylic acid cycle (TCA) and oxidative phosphorylation (OXPHOS) [1]. Beyond their primary role in energy metabolism, mitochondria are highly involved in the pathogenesis of cardiometabolic diseases (CMD), including diabetes mellitus (DM), hypertension, dyslipidemia, and coronary heart disease, whose prevalence gradually increases, with the absence of sufficient physical activity, excessive drinking, and smoking [2].

Mitochondria dynamically reshape, having high plasticity, through fusion and fission cycles to meet the cell’s energy needs, by changing their shape, distribution, and size [3]. The dynamic balance of mitochondrial fission and fusion is vital for ensuring mitochondrial function, particularly when cells encounter metabolic or environmental stress, since fusion helps maintain mitochondrial function by mixing mitochondrial contents, optimizing ATP production, and fission is essential for removing damaged mitochondria and facilitating mitophagy [4]. An imbalance between mitochondrial fusion and fission causes mitochondrial fragmentation, dysfunctional oxidative phosphorylation, and increased ROS generation—characteristic markers of CMDs [5]. Mitochondrial dynamics show an imbalance in DM, atherosclerosis, and hypertension, with impaired fusion/fission balance, defective mitophagy, excessive mitochondrial fragmentation and oxidative stress presented in patients with CMDs [5]. Proteins involved in mitochondrial dynamics such as DRP1 (dynamin-related protein 1), MFN1/2 (mitofusin 1/2), and OPA1 (optic atrophy protein 1) regulate the balance between mitochondrial fission and fusion. DRP1-mediated fission is critical for the removal of damaged mitochondria via mitophagy, but excessive activity contributes to mitochondrial fragmentation and metabolic inflexibility. Reduced MFN2 expression, as observed in hypertensive and diabetic models, impairs mitochondrial connectivity and energy efficiency. OPA1, which mediates inner mitochondrial membrane fusion, is essential for maintaining cristae integrity and respiratory capacity [6,7]. Dysregulation of these dynamics leads to excessive reactive oxygen species (ROS) production, impaired mitochondrial membrane potential, and defective ATP synthesis, promoting disease development and progression [8,9,10].

Mitochondrial dysfunction in cardiometabolic diseases can arise from two major sources: primary mitochondrial defects and secondary mitochondrial dysfunction. Primary defects are often genetic in origin, involving mutations in mitochondrial or nuclear DNA that encode mitochondrial proteins [11]. These hereditary conditions can lead to systemic mitochondrial disorders that may predispose individuals to cardiometabolic complications. In contrast, secondary dysfunction arises due to external metabolic stressors such as chronic overnutrition, sedentary behavior, or oxidative stress, which compromise mitochondrial bioenergetics and signaling [12]. Understanding these distinctions is essential to dissecting the mechanistic complexity and to developing targeted therapeutic strategies.

With the rising prevalence of cardiometabolic diseases projected to increase significantly in the coming years, this narrative review aims to explore the intricate relationship between mitochondrial dysfunction and CMDs. Research for this review was conducted through a comprehensive search of the PubMed and Scopus databases, focusing on the most recent and cutting-edge evidence from observational studies, randomized controlled trials, and clinical investigations; these emerging data shed light on future therapeutic approaches.

## 2. Mitochondrial Dysfunction and Diabetes Mellitus

Type 1 diabetes mellitus (T1DM) is a chronic autoimmune disorder marked by T-cell-mediated destruction of pancreatic β-cells, leading to impaired insulin secretion in the pancreatic islets of Langerhans [13,14]. As the disease progresses, the number of damaged pancreatic β-cells exceeds 75%, resulting in β-cell dysfunction, triggering hyperglycemia and the subsequent need for insulin administration [13]. The biosynthesis and exocytosis of insulin are dependent on mitochondrial adenosine-5′ triphosphate (ATP), energy generated by mitochondria [15,16]. Mitochondrial activity, particularly ROS generation, contributes to β-cell apoptosis during the autoimmune-mediated T1DM development [17]. Mice fed a high-fat high-sucrose diet showed increased ROS production, significant mitochondrial dysfunction in skeletal muscle, and higher insulin resistance [18]. In particular, the production of mitochondrial ROS (mtROS) is highly associated with endoplasmic reticulum (ER) stress-mediated β-cell death, with NF-κB signaling acting as a major pathway driving this interaction [19]. In vitro and in vivo studies have shown that myocytes that contain mitochondria-enriched extracellular vesicles exposed to pathogenetic conditions negatively demonstrated impaired insulin signaling and insulin-stimulated glucose uptake, developing further glucose intolerance [20]. Furthermore, the excessive generation of mtROS promotes macrophage polarization from the M2 anti-inflammatory phenotype to the M1 proinflammatory phenotype, leading to the secretion of cytokines including IL-1β, IL-12, and TNF-α, contributing to the progression of autoimmunity and persistent inflammation in pancreatic β-cells [21,22]. Additionally, CD4+ and CD8+ T-cells play a pivotal role in β-cell destruction, since mtROS affects antigen presentation by dendritic cells, by activating mitochondrial apoptosis via perforin–granzyme mechanisms and Fas–Fas ligand interactions [23]. T1DM progression is further enhanced by the excessive release of IL-1β and IL-18 cytokines, which is promoted by the mitochondria-derived damage-associated molecular patterns (DAMPs), such as oxidized mtDNA and activated NLRP3 inflammasome [24]. Enhanced NLRP3 inflammasome activation is associated with aggravated mitochondrial damage, in conditions of Sirtuin 3 (SIRT3) deficiency, which is protective against mitochondrial dysfunction, especially in diabetic mice with cardiomyopathy [25]. Furthermore, the expression of MHC class I and II molecules is upregulated by IFN-γ, enhancing antigen presentation and promoting autoreactive T-cell infiltration into the islets, promoting further β-cell destruction [26]. Mitophagy also plays a crucial role in the regulation of inflammation, since the process of the defective mitochondria is removed, contributing to downregulation of inflammatory signaling pathways and low-grade chronic inflammation, which is pivotal to the progression of T1DM [27]. Mitochondrial dysfunction shifts drive macrophage polarization toward a proinflammatory state, further activating NLRP3 and NF-κB pathways that promote a perpetuating chronic inflammation, increased cell death, and lower endogenous insulin secretion [28].

## 3. Mitochondrial Dysfunction and Subclinical Cardiovascular Disease

### 3.1. Mitochondrial Dysfunction and Arterial Hypertension

Alterations in mitochondrial dynamics have been associated with activation of the sympathetic nervous system, which is a crucial pathogenetic mechanism for the regulation of arterial hypertension [29]. Cardiometabolic-induced hypertension is characterized by the excessive activation of specific signaling pathways, including the renin–angiotensin–aldosterone system (RAAS), Ca^2+^-activated protein phosphatase calcineurin, and the sympathetic nervous system (SNS) [30].

**Figure 1:**
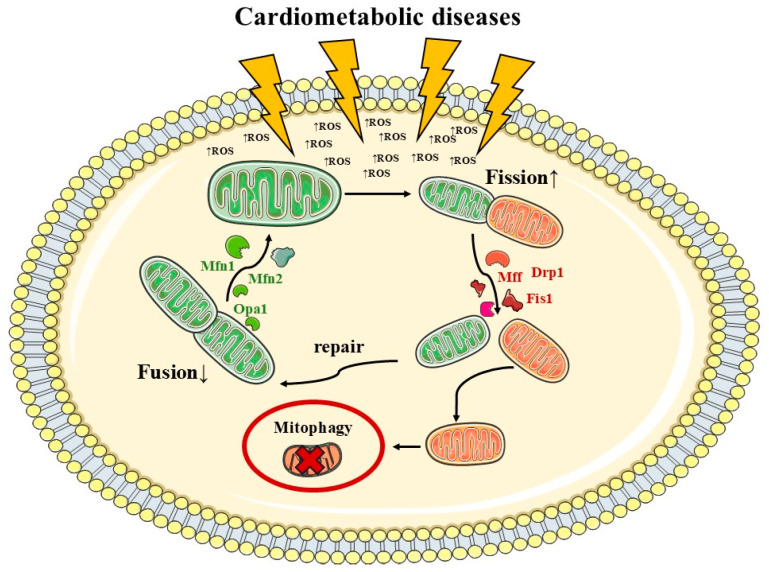
Cardiometabolic diseases’ impact on mitochondrial dynamics. Mitochondrial dynamics are highly affected by oxidative stress and metabolic impairments, causing an imbalance between fusion and fission. Pathophysiology caused by excessive (↑) fission and reduced (↓) fusion. Mitochondrial fission is regulated by proteins such as dynamin-related protein 1 (Drp1), mitochondrial fission factor (Mff), and mitochondrial fission 1 protein (Fis1), and fusion is orchestrated by the mitofusin-1 (Mfn1), Mfn2, and optic atrophy protein 1 (Opa1).

Heart function is dependent on mitochondrial activity, as cardiomyocytes are characterized by a high mitochondrial density, likely due to their need for a continuous and substantial supply of ATP to support their repetitive contractions and the activity of various ion transporters [31]. Hypertension appears to be promoted by cardiomyocyte and cardiac hypertrophy, since increased left ventricular (LV) pressure and volume overload increase afterload, requiring the heart to generate greater force to pump blood [32]. Changes in mitochondrial fusion and fission processes have been linked to various pathological heart conditions [33,34]. In a recent study of cultured neonatal rat cardiomyocytes, epinephrine administration promoted mitochondrial fission, decreased mitochondrial mean volume, and increased the number of mitochondria per cell, showing that increased SNS activation promotes mitochondrial dysfunction [35]. Moreover, a shift towards increased mitochondrial fragmentation was reported in studies in which phenylephrine was administered to induce cardiac hypertrophy [36]. Furthermore, excessive phenylephrine and SNS activation induced a decrease in messenger RNA (mRNA) levels of the mitochondrial fusion protein MFN2 in hypertensive rat models, which demonstrates the pivotal role of mitochondrial dysregulation in arterial hypertension [36]. Moreover, in human umbilical vein endothelial cells, exposure to angiotensin II showed an increased mitochondrial permeability transition pore opening, elevating ROS production, which demonstrates the pivotal role of endothelial mitochondria in hypertension development [37]. Another study, in which mice infused with angiotensin II significantly attenuated the development of hypertension after treatment with the mitochondrial fission inhibitor factors, showed that excessive mitochondrial fission contributes to hypertension [37]. Notably, in hypertension, mitochondrial oxidative damage is induced by the activation of angiotensin (Ang) II, leading to reduced endothelial nitric oxide (NO) levels and increased vascular oxidative stress [38]. Furthermore, Ang II-mediated protein kinase-C activity results in excess production of O^2−^ and H_2_O_2_ that primarily leads to increased ROS production [39]. Mitochondrial quality control (MQC) mechanisms also play a catalytic role in the angiogenic response of endothelial cells to vascular endothelial growth factor (VEGF), since the knock-down of Mitofusin 1 (MFN1) is involved in mitochondrial fusion [40]. Another mechanism that regulates hypertension is mitophagy, resulting in reduced inflammation and ROS production [41]. Hypertensive models show impaired ATG-5-mediated mitophagy during Ang II-induced hypertension and impaired mitochondrial dynamics, respectively, such as reduced mitochondrial mass and structure, mitochondrial size, and osmotic swelling, which disrupts the mitochondrial OXPHOS efficiency [42]. As a result, mitochondrial dysfunction is highly involved in the pathogenesis of hypertension through several pathways that seem to affect mitochondrial dynamics, leading to increased oxidative stress, endothelial dysfunction, and cardiomyocyte hypertrophy (Figure 1).

### 3.2. Mitochondrial Dysfunction and Endothelial Dysfunction

The endothelium plays a crucial role in the vasculature, since it modulates vascular homeostasis by regulating the vascular tone and the vascular permeability of anti-inflammatory, antioxidant, anti-proliferative and anti-thrombotic factors [43]. Endothelial dysfunction and cardiometabolic diseases are interconnected in a bidirectional manner.

Mitochondria are essential for ATP production, cellular metabolic control, and regulation of apoptosis [44]. Their role in maintaining a balance between the production of NO and the concentration of Ca^2+^ is pivotal, since it facilitates the release of NO through the activation of eNOS [45]. Moreover, endothelial homeostasis is severely disrupted by the toxic byproducts of aerobic metabolism, ROS, which are mainly produced by mitochondria [46]. Disturbances in Ca^2+^ balance, resulting in ROS overproduction, have been associated with the opening of the membrane permeability transition pore (mPTP) and disruption in vascular permeability [47]. Moreover, intracellular Ca^2+^ signaling is essential for controlling the vasomotor activity of vascular smooth muscle cells (VSMCs). Mitochondria are key regulators of intracellular calcium levels through their ability to take up and recycle Ca^2^⁺ [48]. Evidence from preclinical research suggests that mitochondrial Ca^2+^ dynamics influence both the function and viability of vascular cells. In endothelial cells, hexokinase influences mitochondrial Ca^2^⁺ balance by blocking the voltage-dependent anion channel. In coronary endothelial cells, hyperglycemia-induced hexokinase upregulation decreases mitochondrial Ca^2^⁺ and limits ROS production [49]. These disruptions can cause permanent damage to the endothelial cellular architecture and trigger the process of apoptosis or programmed cell death [50].

Furthermore, the generation of ROS and oxidative stress facilitates leukocyte adhesion molecules. Mitochondrial-induced endothelial dysfunction results in excessive ROS production and NADPH oxidase (NOX) and e-NOS dysregulation, along with a decline in antioxidant defense mechanisms [51]. The overactivation of NOX generates excessive ROS, being a contributing factor to endothelial cell dysfunction [52]. Additionally, mitochondria-derived ROS, which can take place at complex I or complex III, are essential in regulating cell signaling pathways in endothelial cells. More specifically:

- Complex I (NADH: ubiquinone oxidoreductase) dysfunction often leads to impaired electron flow and increased electron leakage to oxygen, generating superoxide radicals. This dysfunction is particularly relevant in insulin-resistant states, where excessive ROS can impair insulin receptor signaling and reduce glucose uptake in peripheral tissues [18,53].
- Complex II (succinate dehydrogenase) contributes less directly to ROS production but plays a key role in linking the TCA cycle to the electron transport chain. Mutations or reduced activity can disrupt both energy production and metabolic flexibility [53,54].
- Complex III (cytochrome bc1 complex) can also leak electrons to oxygen, especially during reverse electron transport, which is a major source of ROS in endothelial cells under hyperglycemic conditions. This contributes to vascular oxidative stress, endothelial dysfunction, and atherosclerosis [55].
- Complex IV (cytochrome c oxidase) deficiency leads to the incomplete reduction of oxygen, impaired ATP synthesis, and further ROS accumulation, which has been linked to impaired myocardial energetics and contractility in heart failure [56].
- Complex V (ATP synthase) dysfunction primarily affects ATP production, but emerging evidence suggests it also modulates mitochondrial membrane potential and may influence ROS indirectly [57].

The electron transport chain serves as the primary generator of mitochondrial reactive oxygen species (mtROS) [58]. Excessive mtROS release has been proven to contribute both to acute and chronic impairment of endothelial function by altering the signaling of key proteins involved in vascular regulation [59,60]. Notably, endothelial cells are exposed not only to endogenous oxidative species but also to numerous exogenous sources of reactive species, which exacerbate ROS-induced endothelial dysfunction [54]. During atherosclerosis, activated neutrophils release large quantities of ROS in areas where the endothelium is damaged [54]. Animal studies have demonstrated that, in aged mice, treatment with mitochondrial-targeted antioxidants improved vascular endothelial function [61].

Mitochondria also have a catalytic role in the regulation of endogenous mitochondrial antioxidant defense. One mechanism of maintaining redox homeostasis and excessive mitochondria-derived ROS is superoxide dismutase (SOD) isozymes, which catalyze the conversion of O_2_^−^ into H_2_O_2_ and increase the NO bioavailability [62]. In particular, SOD2 is a key endogenous mitochondrial antioxidant mechanism when considering vascular function and enhanced NO bioavailability, due to its vasodilatory properties [63]. Sirtuin-3 (SIRT-3), a mitochondrial localized deacetylase, seems to modulate SOD2 activity and the antioxidant defense system, respectively [64].

### 3.3. Mitochondrial Dysfunction and Atherosclerosis

The mitochondrial respiratory chain efficiently produces energy, with over 98% of electrons used for energy and only 1–2% generating ROS. Oxidative stress occurs when the organism cannot neutralize the overproduction of ROS and reactive nitrogen species (RNS) [65]. Metabolic disease-associated mitochondrial oxidative stress results from an imbalance between elevated ROS/RNS generation and inadequate antioxidant protection [53]. Elevated oxidative stress, resulting from impaired antioxidant mechanisms, can facilitate the progression of atherosclerotic disease.

Moreover, oxidative stress is highly involved in atherogenesis by the oxidation of low-density lipoprotein (LDL) resulting in oxLDL [55,66]. Alterations in the structure of lipoprotein particles can affect lipid, carbohydrate, and protein sections of LDL particles, resulting in modifications of density, size, and other LDL-like particles such as small dense LDL (sdLDL), characterized by reduced antioxidant activity [67]. Such particles remain in the arterial wall for an extended period due to interactions with tissue elements like glycans, leading to a buildup of areas prone to atherosclerosis [67,68]. This prolonged oxidative stress contributes to the development of inflammation in endothelial cells, whose role is crucial for the process of atherosclerosis [66,69].

Furthermore, mitochondrial DNA (mtDNA) is highly affected by ROS/RNS-induced damage, as a result of prolonged low-grade inflammation [70]. The absence of histones and limited repair mechanisms make mtDNA more vulnerable to damage from ROS, contributing to oxidative damage to the respiratory chain and lipid peroxidation [71,72]. Impaired mitochondrial genome function affects mitochondrial physiology and ATP production, contributing to increased ROS levels and enhanced mitochondrial apoptosis [73].

**Figure 2:**
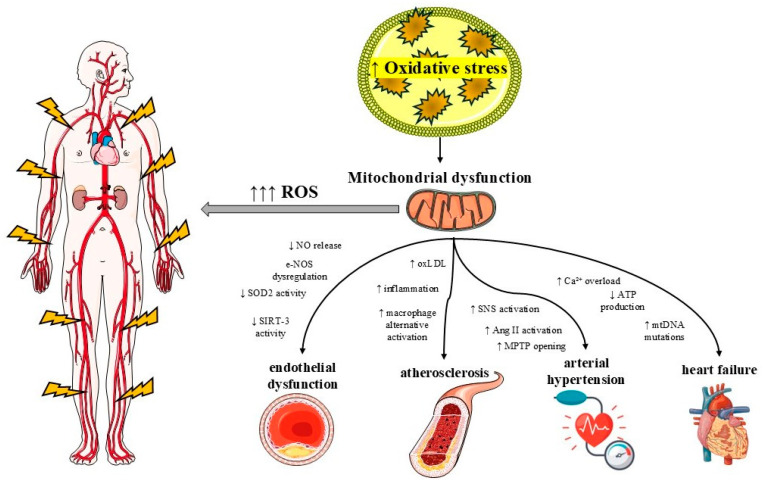
Overview of the vascular consequences of mitochondrial dysfunction. Mitochondrial impairment contributes to endothelial dysfunction via reduced nitric oxide (NO) bioavailability, endothelial nitric oxide synthase (eNOS) dysregulation, decreased superoxide dismutase 2 (SOD2) activity, and diminished sirtuin-3 (SIRT3) function. It promotes atherosclerosis by enhancing oxidized LDL (oxLDL) accumulation, sustaining chronic low-grade inflammation, and promoting alternative macrophage activation. In the pathogenesis of arterial hypertension, mitochondrial dysfunction leads to increased sympathetic nervous system (SNS) activity, heightened angiotensin II (Ang II) signaling, and increased mitochondrial permeability transition pore (MPTP) opening. In heart failure, it contributes through calcium (Ca^2^⁺) overload, accumulation of mitochondrial DNA (mtDNA) mutations, and impaired ATP production.

The role of macrophages is also pivotal in the progression of atherosclerotic lesions, since their activation either classically (M1) or activated macrophages (M2) contributes to destabilization of the atherosclerotic fibrous cap region and lipid accumulation in the atheromatic plaques [74]. Mitochondrial oxidative metabolism supports macrophage alternative activation, with both M1 and M2 reducing oxygen consumption and damaging mtDNA [75]. M1 macrophages produce large numbers of proinflammatory cytokines such as TNF-α, IL-1β, IL-6, IL-12, IL-18, and IL-23, which is induced by their polarization and is highly affected by mitochondrial ROS above physiological levels [76,77]. Several mutations in mtDNA have been found to contribute to the proinflammatory response in monocytes, changing the activation of monocyte-derived macrophages through mitochondrial dysfunction in atherosclerosis [78] (Figure 2).

### 3.4. Mitochondrial Dysfunction and Clinical Cardiovascular Disease

The cardiomyocyte mitochondria are divided into two distinct populations: one adjacent to the sarcolemma and the other enclosed within the contractile apparatus [79]. In cardiac muscle cells, both mitochondrial groups maintain electrical connectivity, allowing the transfer of electrical signals between mitochondria [80]. The mitochondrial structure is dynamically controlled by fusion and fission proteins, responding to changes in cardiac pathology [81]. For instance, giant mitochondria are observed in some cardiomyopathies and in response to dietary, drug, or toxin exposure [82]. Moreover, mitochondrial biochemical alterations are significant contributors to heart failure [83].

Ischemic myocardial injury caused by an abrupt coronary occlusion triggers ischemic injury, leading to tissue hypoxia and ATP depletion [65]. Hypoxia from impaired blood flow lowers oxidative phosphorylation, decreases cellular ATP levels, and leads to mitochondrial membrane depolarization [84]. Moreover, ischemia-induced hypoxia causes a switch to anaerobic glycolysis in the myocardium, which lowers intracellular pH due to lactate buildup, a byproduct of anaerobic respiration [85]. Cardiac ischemia–reperfusion injury reintroduction to oxygen-rich blood initiates an uncontrolled ROS-driven response, worsening necrosis for up to three days post-reperfusion [86]. Mitochondrial damage during ischemia-reperfusion injury causes a mitochondrial Ca^2+^ overload and unregulated ROS production, which leads to mitochondrial permeability transition pore opening and subsequent cell death [87]. Notably, the heart has low ATP levels due to its high demand and rapid turnover of ATP. Cellular ATP is primarily produced by oxidative phosphorylation (OXPHOS), a process driven by the electron transport chain on the inner mitochondrial membrane [88]. Decreased ATP production and impaired OXPHOS in the ischemic heart lead to a decline in cardiac performance [89].

Moreover, mitochondria are essential not only for ATP generation but also for metabolism, Ca^2+^ homeostasis, lipid synthesis, and redox regulation in the heart [90]. Changes in metabolism can lead to heterogeneity in heart failure presentations and progression. Heart failure with preserved ejection fraction (HFpEF) involves oxidative stress as a key regulator, triggering inflammation, fibrosis, and altered Ca^2+^ homeostasis [91]. Mitochondrial impairment and citric acid cycle abnormalities in diastolic HF correlate with protein hyperacetylation, a process reversible with nicotinamide riboside [92]. At the core of mitochondrial metabolism lies the tricarboxylic acid (TCA) cycle. In particular, in insulin-resistant states or diabetic hearts, there is increased reliance on fatty acid oxidation at the expense of glucose oxidation, leading to a reduced efficiency of the TCA cycle and ATP yield per oxygen molecule consumed, thus promoting metabolic stress [93]. Impaired TCA cycle flux leads to accumulation of metabolic intermediates and electron carriers, contributing to oxidative damage, inflammation, and endothelial dysfunction in both the heart and vasculature [94]. In HFpEF, altered TCA cycle enzyme activity has been linked to protein hyperacetylation that impairs the function of key TCA cycle enzymes, further exacerbating metabolic inefficiency [95]. Metabolic inflexibility and mitochondrial stress further promote the secretion of proinflammatory cytokines and profibrotic mediators, contributing to adverse cardiac remodeling. Furthermore, impaired Ca^2^⁺ regulation is a key characteristic of heart failure. The impact of mitochondrial Ca^2+^ dynamics in heart failure remains debated, with some evidence suggesting mitochondrial Ca^2^⁺ overload is damaging, while other studies indicate potential benefits [96,97,98]. The mitochondrial Ca^2^⁺ uniporter (MCU) facilitates Ca^2^⁺ uptake, while the Na⁺/Ca^2^⁺ exchanger (NCLX) mediates efflux, both playing crucial roles in mitochondrial Ca^2^⁺ homeostasis [99]. Studies suggest that mitochondrial Ca^2^⁺ overload can be detrimental, contributing to oxidative stress and cardiomyocyte death, but in some models, increased mitochondrial Ca^2^⁺ enhances energy production, indicating a complex and context-dependent role in heart failure [100,101,102]. Mitochondrial ROS (mtROS) also play a dual role in heart failure, contributing to both physiological and pathological processes. Excessive mtROS in the failing heart lead to mitochondrial damage, opening of the mitochondrial permeability transition pore (mPTP), and cell death, while impairing mitochondrial function and biogenesis [103,104]. Antioxidant systems, such as peroxiredoxin (Prx) and glutathione peroxidase (Gpx), help mitigate ROS damage, but dysfunction in these systems, along with mitochondrial NADPH supply issues, exacerbates oxidative stress, impairs ATP production, and worsens heart failure [57,105,106]. In addition, mtROS contribute to a variety of cellular dysfunctions, including protein and lipid damage, inflammation, and impaired mitochondrial signaling [107]. Finally, mitochondrial DNA (mtDNA) is crucial in heart failure (HF), with studies showing a >40% reduction in mtDNA content and impaired replication in failing hearts, hindering mitochondrial biogenesis [56,108]. MtDNA is uniquely vulnerable to damage, and its repair capacity is significantly limited compared to nuclear DNA, due to the lack of protective histones, which, in the nucleus, serve to protect DNA from oxidative damage and regulate transcription [109]. The mitochondrial inner membrane is a primary site of ROS production. mtDNA lies in close proximity to the electron transport chain, increasing the risk of oxidative lesions [109]. A 30-year study, the Atherosclerosis Risk in Communities, revealed an inverse relationship between mtDNA copy number and heart failure risk [110]. Due to the lack of histones and limited epigenetic control, mtDNA is exclusively impacted by oxidative stress, unlike the nuclear genome [111]. These findings show that several mitochondrial mechanisms have been identified that contribute to heart failure before ATP depletion occurs (Table 1).

## 4. Other Mitochondrial Factors and Cardiometabolic Disease

### 4.1. Mitochondrial Open Reading Frame of the 12S rRNA Type-c (MOTS-c)

The mitochondrial open reading frame of the 12S rRNA type-c (MOTS-c) consists of 16 amino acids forming an α-helical structure [123]. In animal models, MOTS-c enhances insulin sensitivity in skeletal muscle while protecting against diet-induced obesity and insulin resistance [124]. Studies have shown that MOTS-c levels were significantly lower in young obese males, an inverse correlation with insulin resistance in lean individuals, and found to be lower in patients with type 1 and type 2 diabetes [125,126]. Furthermore, MOTS-c shows protective effects in CVD, since studies suggest improving cardiac function and pathological ventricular remodeling by activating pathways such as AMPK that mimic the benefits of aerobic exercise, though its precise target in cardiomyocytes remains unclear [127]. In diabetic rats, MOTS-c demonstrated protection against myocardial mitochondrial damage and cardiac function, with beneficial effects on diabetic cardiomyopathy [128]. Moreover, MOTS-c has anti-inflammatory effects by regulating cytokine levels through AMPK activation and increasing anti-inflammatory cytokines while decreasing proinflammatory cytokines [129,130]. MOTS-c is the first peptide encoded that has been included in clinical trials for its protective effects, showing the pivotal role of mitochondria in therapeutics and drug targets [131].

### 4.2. Damage-Associated Molecular Patterns (DAMPs)

Damage-associated molecular patterns (DAMPs) are internal danger signals that are released from cells undergoing damage or death, which activate the innate immune response by binding to pattern recognition receptors (PRRs) [132]. In DM patients, chronic hyperglycemia through IL-1β secretion from various cell types, including β cells, triggers a strong danger signal that impairs insulin secretion, promotes β-cell death, and contributes to insulin resistance [133]. The regulation of proinflammatory cytokine processing and secretion is primarily controlled by inflammasomes in cardiometabolic diseases, especially from nucleotide-binding domain and leucine-rich repeat protein 3 (NLRP3) inflammasome, which plays a crucial role in T2DM [134]. High glucose levels activate NLRP3 inflammasomes, which exacerbate β-cell dysfunction and insulin resistance in both T1DM and T2DM, enhancing inflammation and impairing insulin signaling [135]. Moreover, DAMPs enhance endothelial dysfunction, since they contribute to the promotion of foam cell formation and lipid accumulation, affecting atherosclerotic plaques and vascular inflammation [136]. Mitochondrial DNA (mtDNA) and other mitochondrial components are recognized as mitochondrial damage-associated molecular patterns (mtDAMPs) that lead to the progression of inflammation and atherosclerotic plaque formation, contributing to atherosclerosis, insulin resistance, and hypertension [133]. Medium-chain acylcarnitines (MCACs) and long-chain acylcarnitines (LCACs) predict major adverse cardiovascular events (MACE) in type 2 diabetes patients (TECOS/EXSCEL cohorts, *p* < 0.01). Elevated dicarboxylacylcarnitines associate with 32% increased MACE risk after multivariable adjustment (HR 1.32, 95% CI 1.12–1.55) [137]. Finally, several other proteins and mitochondrial elements have been related to mitochondrial dynamics, quality control, and signaling, and they play pivotal roles in the development and progression of cardiometabolic diseases (Table 2).

## 5. Potential Treatment Targets

Several therapeutic agents have been developed to target mitochondrial dysfunction. MitoQ, a mitochondria-targeted antioxidant, has shown efficacy in reducing vascular oxidative stress in both animal models and early-phase clinical trials [143]. NAD+ precursors such as nicotinamide riboside (NR) and nicotinamide mononucleotide (NMN) improve mitochondrial function by enhancing SIRT1/3 activity, boosting mitochondrial biogenesis and antioxidant defense. However, limitations such as short half-lives, tissue-specific bioavailability, and variability in clinical responses necessitate further investigation through randomized controlled trials.

In addition to MitoQ and NAD+ boosters, other agents like elamipretide (SS-31) have gained attention for stabilizing cardiolipin and improving mitochondrial respiration in failing hearts [144]. Clinical trials have demonstrated that elamipretide can enhance left ventricular function and mitochondrial efficiency in patients with heart failure with reduced ejection fraction (HFrEF) [145]. Moreover, natural compounds such as resveratrol and coenzyme Q10 have shown promise in modulating mitochondrial oxidative stress and inflammation, although the results have been inconsistent in larger clinical studies [146,147].

Gene therapy approaches and mitochondrial transplantation are emerging frontiers. For example, adenoviral-mediated overexpression of PGC-1α and TFAM in preclinical models has improved mitochondrial biogenesis and attenuated cardiac remodeling [148]. While still experimental, mitochondrial transfer techniques have been successfully applied in preclinical settings to rescue ischemic tissues and may offer therapeutic potential in the context of CMDs [149].

## 6. Limitations of the Review

This review is primarily based on the published literature and does not include a systematic meta-analysis of the data. While effort was made to include high-quality human and preclinical studies, there may be publication bias favoring positive findings. In addition, many mechanistic insights are derived from animal models, which may not fully replicate human cardiometabolic disease pathology. The rapidly evolving nature of mitochondrial therapeutics means that some recent advances may not be comprehensively captured.

## 7. Conclusions

Mitochondrial dysfunction plays a fundamental role in the development and progression of CMDs by regulating oxidative stress, inflammatory responses, and cellular homeostasis, with disruptions in mitochondrial dynamics also contributing to several complications. Excessive mtROS and RNS production, overproduction of proinflammatory cytokines, reduced endothelial nitric oxide secretion, and mtDNA alterations are crucial for the imbalances in mitochondrial homeostasis, whose future research may pave the way for innovative strategies to combat CMDs and improve long-term cardiometabolic health. Future research should address key knowledge gaps, including the precise role of mitochondrial calcium homeostasis, tissue-specific responses to mitochondrial stress, and the bidirectional crosstalk between mitochondria and nuclear epigenetics in CMDs. Moreover, integrating mitochondrial profiling into precision medicine frameworks could enable tailored therapeutic approaches based on mitochondrial phenotypes. Large-scale longitudinal human studies incorporating mitochondrial biomarkers are needed to validate their utility in risk stratification and treatment monitoring.

## Figures and Tables

**Table 1:** Mechanisms by which mitochondrial dysfunction affects cardiometabolic diseases.

Disease/Condition	Mitochondrial Mechanism	Pathophysiological Effects
Diabetes Mellitus (DM)	Impaired mitochondrial ATP production	Reduces insulin biosynthesis and secretion [16]
	Excessive mitochondrial ROS (mtROS) production	Induces β-cell apoptosis via ER stress and NF-κB activation [112]
	MtROS-mediated antigen presentation by dendritic cells	Enhances β-cell destruction via CD4+ and CD8+ T-cell activation [113]
	Impaired mitophagy	Increases β-cell death and chronic low-grade inflammation [114]
	Mitochondria-derived DAMPs (oxidized mtDNA, NLRP3 activation)	Induces proinflammatory cytokines (IL-1β, IL-18) [115]
Endothelial Dysfunction	Mitochondrial NO production and Ca^2^⁺ balance dysregulation	Reduces endothelial-dependent vasodilation [45]
	mtROS overproduction due to Ca^2^⁺ imbalance and mPTP opening	Disrupts vascular permeability and triggers endothelial apoptosis [116]
	Excessive NADPH oxidase (NOX) activation	Increases oxidative stress, impairing endothelial function [51]
	Impaired mitochondrial antioxidant defenses (SOD2, SIRT3 dysfunction)	Enhances vascular inflammation and oxidative stress [63,64]
Atherosclerosis	Increased mtROS production	Oxidizes LDL, leading to small dense LDL (sdLDL) formation and foam cell generation [117]
	mtDNA oxidative damage	Impairs mitochondrial respiration and enhances chronic inflammation [118]
	mtROS-mediated macrophage polarization (M1 activation)	Increases proinflammatory cytokines (TNF-α, IL-1β, IL-6, IL-12, IL-18, IL-23) [119]
	mtDNA mutations	Enhances monocyte activation and atherogenesis [120]
Heart Failure	Ischemia-induced mitochondrial dysfunction	Reduces ATP production and impairs cardiac contractility [85]
	Hypoxia-mediated mitochondrial depolarization	Switches metabolism to anaerobic glycolysis, decreasing ATP levels [85]
	Ischemia-reperfusion injury-induced ROS overproduction.	Causes mitochondrial Ca^2^⁺ overload, mPTP opening, and cardiomyocyte death [103,104]
	Mitochondrial Ca^2^⁺ dysregulation	Impairs cardiac energy production, exacerbating HF progression [100]
	Decreased mtDNA copy number and impaired replication	Lowers mitochondrial biogenesis and cardiac function [121,122]

ER: endoplasmic reticulum, NF-Κb: Nuclear factor kappa-light-chain-enhancer of activated B cells.

**Table 2:** Proteins and mitochondrial elements involved in mitochondrial dysfunction in cardiometabolic diseases.

Molecule	Main Function	Role in Cardiometabolic Diseases (CMDs)
UCP2 (Uncoupling Protein 2)	Reduces mitochondrial membrane potential and reactive oxygen species (ROS) production.	Overexpression of UCP2 may decrease ATP production, impairing insulin secretion from pancreatic β-cells [138]
DRP1 (Dynamin-Related Protein 1)	Splits mitochondria, important for mitochondrial turnover and quality control.	Excessive fission (DRP1 hyperactivation) leads to fragmented mitochondria, mitochondrial dysfunction, and increased apoptosis, especially in cardiomyocytes and vascular cells [139]
PARKIN (E3 Ubiquitin Ligase)	Mediates mitophagy—selective removal of damaged mitochondria—via the PINK1-PARKIN pathway.	Accumulation of dysfunctional mitochondria contributes to oxidative stress, inflammation, and cell death in metabolic tissues [140]
mtDNA Haplogroups	Influence mitochondrial function, ROS production, and bioenergetics.	Affect OXPHOS efficiency, adaptive thermogenesis, and susceptibility to oxidative stress [141]
Mitochondrial Open Reading Frame of the 12S rRNA type-c (MOTS-c)	Regulates metabolism and stress responses.	Enhances insulin sensitivity, promotes glucose uptake, and suppresses inflammation [128]
DAMPs (Damage-Associated Molecular Patterns)	Is released from stressed or dying cells.	Contribute to chronic low-grade inflammation [142]

OXPHOS: Oxidative phosphorylation.

## Data Availability

Not applicable.
